# Distribution of depression, somatization and pain-related impairment in patients with chronic temporomandibular disorders

**DOI:** 10.1590/1678-7757-2018-0210

**Published:** 2019-01-07

**Authors:** Giancarlo De La Torre Canales, Luca Guarda-Nardini, Célia Marisa Rizzatti-Barbosa, Paulo César Rodrigues Conti, Daniele Manfredini

**Affiliations:** 1 Universidade de São Paulo, Faculdade de Odontologia de Bauru, Departamento de Prótese, Bauru, São Paulo, Brasil.; 2 Università di Padova, Dipartimento di Chirurgia Maxillo-Facciale, Padova, Italia.; 3 Universidade Estadual de Campinas, Faculdade de Odontologia de Piracicaba, Departamento de Prótese e Periodontia, Piracicaba, São Paulo, Brasil.; 4 Università di Siena, Scuola di Odontoiatria, Siena, Italia.

**Keywords:** Temporomandibular disorders, Psychosocial impact, Depression

## Abstract

**Objective:**

the aim of this study was to describe the frequency of psychosocial diagnoses in a large sample of patients attending a tertiary clinic for treatment of temporomandibular disorders (TMD).

**Material and Methods:**

six hundred and ninety-one patients who sought treatment for pain-related TMD were selected. Chronic pain-related disability (Graded Chronic Pain Scale, GCPS), depression [Symptoms Checklist-90 (SCL-90) scale for depression, DEP] and somatization levels (SCL-90 scale for non-specific physical symptoms, SOM) were evaluated through the Research Diagnostic Criteria for TMD (RDC/TMD) Axis II psychosocial assessment; TMD diagnoses were based on the Axis I criteria.

**Results:**

the majority of patients presented a low disability or no disability at all, with only a small portion of individuals showing a severely limiting, high disability pain-related impairment (4.3%). On the other hand, abnormal scores of depression and somatization were high, with almost half of the individuals having moderate-to-severe levels of depression and three-fourths presenting moderate-to-severe levels of somatization. The prevalence of high pain-related disability (GCPS grades III or IV), severe/moderate depression and somatization was 14.3%, 44% and 74.1% respectively. Gender differences in scores of SCL-DEP (p=0.031) and SCL-SOM (p=0.001) scales were signficant, with females presenting the highest percentage of abnormal values.

**Conclusion:**

patients with TMD frequently present an emotional profile with low disability, high intensity pain-related impairment, and high to moderate levels of somatization and depression. Therefore, given the importance of psychosocial issues at the prognostic level, it is recommended that clinical trials on TMD treatment include an evaluation of patients’ psychosocial profiles.

## Introduction

Several studies have reported that patients with chronic pain conditions show high psychosocial impairment compared with pain-free control groups.^1^ ^,^ ^2^ These psychosocial variables are associated with poorer pain-related adjustment among patients with chronic pain.^3^ Similar results have also been reported for patients with painful temporomandibular disorders (TMD) (i.e., myofascial pain, arthralgia, arthritis), who showed higher psychosocial impairment than TMD-free individuals.^4^ ^,^ ^5^

Based on such observations, theories on the etiology of TMDs and its implications for treatment have progressively embraced the importance of a comprehensive biological and psychosocial assessment^6^ and TMDs are now viewed as a complex disorder resulting from an interplay of causes, including multiple genetic and environmental domains.^7^ Psychological impairment is associated with greater severity and persistence of TMD-related clinical symptoms,^7^ which affect approximately 10% of the population, with a higher prevalence in females.^8^

The Research Diagnostic Criteria for Temporomandibular Disorders (RDC/TMD) Axis II^9^ was specifically designed for a thorough psychosocial assessment, allowing evaluation of the severity of chronic pain and the levels of depression and somatization in TMD patients. The revised and updated version, now called Diagnostic Criteria for TMD (DC/TMD),^10^ widened the usefulness of the instruments to the clinical setting, thanks to a refinement of Axis I (i.e., physical) algorithms and the addition of some Axis II measures. Nonetheless, the core features of the original Axis II that have been used for years to collect psychosocial data on TMD patients as part of the RDC/TMD guidelines are still useful tools to share epidemiological data among the different research groups, as well as to characterize behavioral features in clinical settings.

In the light of recent observations that only a few articles have reported Axis II findings, basically limiting the construct of a biopsychosocial model for pain, further studies are required to improve the knowledge on the epidemiology and prevalence of psychosocial factors in TMD patients.^11^ ^,^ ^12^ Based on that, multicenter studies have been performed to depict the frequency of Axis II findings in TMD patients.^13^ The absence of correlation between Axis I, i.e. the diagnoses of TMD physical symptoms, and Axis II, i.e. the level of psychological and pain-related impairment, has been reported.^14^ Moreover, treatment-seeking behavior seems to be the discriminant factor to differentiate patient and non-patient populations, and psychosocial factors emerged as the main predictor of treatment outcome.^15^

Considering these drawbacks, the paucity of epidemiological data on Axis II is still evident in the TMD literature. Therefore, the aim of this study was to describe the frequency of psychosocial diagnoses in a large sample of patients attending a tertiary TMD clinic to provide an epidemiological basis for future comparisons.

## Material and methods

### Study population

A total of 691 patients (571 women, 120 men; mean age: 42.5 years, range: 18 to 61 years) who sought treatment at the TMD Clinic, Department of Maxillofacial Surgery, University of Padova, Italy, from 2011 to 2015 for pain-related TMD were selected. Exclusion criteria were age less than 18, diagnosis of other orofacial pain disorders, and presence of polyarthritis and/or other rheumatic disease.

### Assessment instruments

Complete examination was carried out according to the Italian version of the RDC/TMD protocol (RDC/TMD Consortium Network). Psychosocial status was assessed by the Axis II questionnaire, which contains specific items for the appraisal of chronic pain severity and of subjective signs and symptoms for levels of depression and somatization.^9^

The Graded Chronic Pain Scale (GCPS)^16^ allows the categorization of pain patients into five levels of pain-related impairment (from 0: no TMD pain in the prior 6 months, to IV: high disability, severely limiting); while the Depression and Somatization scales of the Symptom Checklist 90R (SCL-90R), SCL-DEP and SCL-SOM, respectively,^17^ categorized patients within three groups as normal, moderate, or severe levels.

The frequencies of the different scores for the GCPS, SCL-DEP, and SCL-SOM in the study population were reported by descriptive analysis. Analysis of variance (ANOVA) test was used for age differences between patients with different Axis II ratings, and chi-squared test was used for gender comparisons.

All patients gave their written informed consent to the clinical diagnostic procedures undertaken during the investigation, and the study protocol was approved by the University of Padova's Institutional Review Board.

## Results

GCPS scores showed that most patients were rated as grade I or II, with 42.0% having low disability but high intensity pain-related impairment. Conversely, only 7.9% of patients reported no disability at all (grade 0), and 4.3% showed severely limiting, high disability (grade IV) (Table 1).

**Table 1:** Distribution of GCPS ratings

GCPS Categories	(%)
No disability	7.9
Low disability, low intensity (grade I)	35.4
Low disability, high intensity (grade II)	42
High disability, moderately limiting (grade III)	10.2
High disability, severely limiting (grade IV)	4.3

GCPS: Graded Chronic Pain Scale

Approximately 74.1% of patients showed abnormal values on the SCL-SOM scale, indicating severe (50.9%) or moderate (23.2%) somatization levels. As for the SCL-DEP scale, the percentage of patients with abnormal values was lower (41.0% severe, 3.0% moderate) (Table 2).

**Table 2:** Percentage of patients receiving different scores in the Research Diagnostic Criteria for Temporomandibular Disorders (RDC/TMD) Axis II Depression (SCL-DEP) and Somatization (SCL-SOM) scales

	SCL-DEP	SCL-SOM
Normal	56%	25.8%
Moderate	3%	23.2%
Severe	41%	50.9%

SCL-DEP: Symptom Checklist 90R - Depression; SCL-SOM: Symptom Checklist 90R - Somatization

Age differences between patients with different Axis II scores were not significant (GCPS, p=0.769; SCL-SOM, p=0.592; SCL-DEP, p=0.707). Gender differences in scores of SCL-DEP (p=0.031) and SCL-SOM (p=0.001) scales were signficant, with females presenting the highest percentage of abnormal values. Females had the highest frequency of high pain-related impairment (15.2%) in the GCPS scores, even though most female individuals presented low disability, high intensity pain-related impairment (42.7%) (Table 3). Likewise, females presented the highest frequency of severe impairment in the SCL-DEP and SCL-SOM scales, with 42.6% and 53.4% respectively (Table 4).

**Table 3:** Percentage of genders according to GCPS category

GCPS Scales	Female (%)	Male (%)
No disability	7.5	10
Low disability, low intensity (grade I)	34.5	40
Low disability, high intensity (grade II)	42.7	38.3
High disability, moderately limiting (grade III)	10.6	8.3
High disability, severely limiting (grade IV)	4.5	3.3

GCPS: Graded Chronic Pain Scale.

* p<0.05 for abnormal values

**Table 4:** Percentage of genders allocated according to the severity of Depression (SCL-DEP) and Somatization (SCL-SOM) scales

		Female (%)	Male (%)
SCL-DEP	Normal	55	61.7
	Moderate	2.4*	5.8
	Severe	42.6*	32.5
SCL-SOM	Normal	23.3	38.3
	Moderate	23.3*	23.3
	Severe	53.4*	38.3

SCL-DEP: Symptom Checklist 90R - Depression; SCL-SOM: Symptom Checklist 90R - Somatization.

* p<0.05 for abnormal values

## Discussion

The importance of assessing psychosocial factors in TMD patients has been recognized in the literature, which showed an association between TMD pain and psychological symptoms including depression, somatization, and anxiety.^6^ ^,^ ^14^ To address this issue, the standardized guidelines of RDC/TMD Axis II provide useful assessment tools for psychosocial appraisal of TMD patients and for rating of pain-related impairment, i.e. disability and limitations in an individual's everyday life.^9^ Notwithstanding, only few studies addressed the issue of psychosocial disorders in TMD patients and focused on the description of the entire spectrum of symptoms included in the Axis II evaluation, i.e. both the rating of pain-related impairment and the levels of depression and somatization. This is important in the light of the recently described absence of correlation between physical (i.e., Axis I) and psychosocial findings (i.e., Axis II) with the latter, rather than the former, being the key issue to predict the treatment outcome.^14^ ^,^ ^15^ Thus, approaching TMD epidemiology without taking into account the Axis II limits the construct of the so-called biopsychosocial model of pain.^11^ Therefore, this large-sample investigation reports Axis II findings in a population of TMD patients attending a tertiary clinic to provide a framework for clinicians who could expect patients with different ratings of psychosocial impairment, regardless of the Axis I diagnoses.

In this investigation, based on GCPS scores, the frequencies of the most severe degrees of pain-related impairment were 10% for grade III and 4.3% for grade IV. Available data on the different GCPS categories reported prevalence of 3.1% and 6.3% of high intensity, severely and moderately limiting pain respectively,^18^ which is similar to the findings of this study. This investigation is also in line with other three studies reporting a range of 13% to 21.8% for the two most severe GCPS ratings.^5^ ^,^ ^7^ ^,^ ^14^ This variability of results is likely reflecting differences in patient samples, possibly due to strategies of patient recruitment and referral as well as cultural attitudes towards treatment-seeking behavior. Notwithstanding, it can be confirmed that a minority of patients with TMDs reported high pain-related impairment, and only a very small proportion (4.3% in the present investigation) developed such highly disabling pain leading to severe limitation.

The importance of assessing the levels of pain intensity and pain-related disability evaluated by the GCPS lies in its influence on the clinical decision-making process, i.e. knowing or not such profile is emerging as a factor that affects the prognosis of TMD symptoms. In short, it can be suggested that patients with severe impairment are the worst treatment responders, while those with low impairment seem to have benefit even from “simple” cognitive-behavioral therapy regimen and may take advantage of the positive natural variation of symptoms.^14^ ^,^ ^15^ ^,^ ^19^ In addition, the GCPS has been used to identify groups of patients that may benefit more from cognitive-behavioral approaches.^20^ Thus, it is quite surprising that the number of research on pain-related impairment in TMD patients is not relevant; also, the increase in the diffusion of GCPS in both research and clinical settings is strongly recommended to aid the selection of an appropriate treatment protocol including tailored strategies to address pain-related impairment.^21^ Lack of Axis II records and/or inappropriate interpretation of Axis II findings is a shortcoming that negatively affects the definition of management strategies in clinical settings.

As for the SCL-90R scores, moderate to severe levels of depression and somatization were detected in 44% and 74.1% of patients, respectively. These findings are similar to available data in which the prevalence of depression and somatization was 49% and 69% respectively;^4^ and with data reported by other research groups who conducted similar investigations.^22^ ^-^ ^24^ Based on all these data, it could be suggested that the association between TMD and psychosocial factors is part of a more complex pain-psychopathology association, including at least symptoms of depression and somatization

In TMD patients, somatization severity has been useful to distinguish the perception of the physical intensity of pain, and to evaluate its cognitive and emotional meaning. In addition, several studies have found a relationship between measures for somatization and clinical pain in populations with chronic pain, including chronic TMD patients. In particular, somatization is related to a more widespread pain,^25^ ^,^ ^26^ with the number of coexisting chronic pain conditions,^25^ with complaint of symptoms in the absence of organic disease,^27^ and with TMD treatments outcomes.^28^

Similarly, depression in chronic TMD populations has been associated with several reported pain conditions,^29^ treatment outcomes, altered pain perception and thresholds.^30^ There is considerable comorbidity between the clinical characteristics of affective and somatoform disorders, since many criteria that are associated with depression involve bodily symptoms, behavioral avoidance, and appraisal of events as threatening, thus also being potential signs of somatization.

Selecting behavioral treatments based on psychosocial profiles was shown to be successful in TMD patients,^20^ ^,^ ^28^ ^,^ ^31^ due to the non-negligible portion of individuals with Axis II disorders and their consequent influence on treatment outcomes. Therefore, it is possible that treatment effects for a depressed patient could be enhanced in a different way than for a somatically focused patient by targeting passive behavioral responses to pain and training patients in behavioral and self-regulation exercises, respectively.^32^ Therefore, these findings suggest that, due to the influence of psychosocial factors, clinical trials should consider different types of treatment protocols based also on the psychosocial profile of patients.

This study also assessed the gender distribution of TMD diagnoses regarding Axis II findings. Gender-stratified distribution showed that females presented the highest scores in all three Axis II scales for abnormal values. This result is similar to that reported in a sample of Asian TMD patients,^22^ in which the prevalence of females with abnormal values in the three scales was higher compared to males. Conversely, Rantala^33^ (2004) reported similar abnormal scores between females and males in the depression and somatization scales, which is in contrast with the present findings. Therefore, it is cautionary to suggest that further research is needed to explore how differences in gender, culture, ethnicity, and variations in healthcare provision are possible factors influencing the differential expression of TMD in patients around the world.

This investigation has some shortcomings that could be addressed in future studies. The main limitation is the absence of information on the Axis I diagnosis, which might have given a more complete clinical picture. On the other hand, the absence of correlation with the psychosocial findings has already been shown,^14^ and Axis II is emerging as the most important outcome predictor for treatment purposes.^15^ ^,^ ^21^ As a further note, the inclusion of TMD-free control groups could impact the relative importance of psychosocial impairment in TMD patients with respect to the general population, but it should be remarked that previous case-control studies support a higher Axis II impairment in TMD patients.^4^ Moreover, despite all aforementioned statements about the TMD-psyche relationship and usefulness of the RDC/TMD Axis II scales for the evaluation of depression and somatization symptoms,^26^ it must be remarked that they provide an assessment of clinical characteristics and are not diagnostic of any psychopathology. Based on that, the inclusion of psychologists in the team of caregivers is recommended when such screening tools identify severe Axis II symptoms. Finally, future investigations using the DC-TMD and the additional Axis II tools will help to assess the psychosocial profile of TMD patients in a more comprehensive way.

Thus, to our knowledge, this investigation presented the largest Axis II data collection in a TMD population for future comparison. Methodological issues concerning the size and representativeness (e.g. type of TMD, pain duration) of the study population should be considered for refinement and comparison with future investigations.

## Conclusions

Based on our findings, it can be concluded that patients with TMD frequently present an emotional profile with low disability, high intensity pain-related impairment and with high to moderate levels of somatization and depression. Given the importance of psychosocial issues at the prognostic level, it is recommended that these data are taken as reference standpoint for future comparisons and that clinical trials on TMD treatment include an evaluation of patients’ psychosocial profiles in order to identify pain phenotypes related to the TMD manifestation.
